# Prediction of BRCA Gene Mutation in Breast Cancer Based on Deep Learning and Histopathology Images

**DOI:** 10.3389/fgene.2021.661109

**Published:** 2021-07-20

**Authors:** Xiaoxiao Wang, Chong Zou, Yi Zhang, Xiuqing Li, Chenxi Wang, Fei Ke, Jie Chen, Wei Wang, Dian Wang, Xinyu Xu, Ling Xie, Yifen Zhang

**Affiliations:** ^1^Department of GCP Research Center, Jiangsu Province Hospital of Chinese Medicine, The Affiliated Hospital of Nanjing University of Chinese Medicine, Nanjing, China; ^2^Department of Pathology, Jiangsu Cancer Hospital, Nanjing, China; ^3^Jiangsu Institute of Cancer Research, The Affiliated Cancer Hospital of Nanjing Medical University, Nanjing, China; ^4^Department of Pathology, Jiangsu Province Hospital of Chinese Medicine, The Affiliated Hospital of Nanjing University of Chinese Medicine, Nanjing, China

**Keywords:** breast cancer, BRCA gene, deep learning, artificial intelligence, digital pathology

## Abstract

**Background:**

Breast cancer is one of the most common cancers and the leading cause of death from cancer among women worldwide. The genetic predisposition to breast cancer may be associated with a mutation in particular genes such as gene BRCA1/2. Patients who carry a germline pathogenic mutation in BRCA1/2 genes have a significantly increased risk of developing breast cancer and might benefit from targeted therapy. However, genetic testing is time consuming and costly. This study aims to predict the risk of gBRCA mutation by using the whole-slide pathology features of breast cancer H&E stains and the patients’ gBRCA mutation status.

**Methods:**

In this study, we trained a deep convolutional neural network (CNN) of ResNet on whole-slide images (WSIs) to predict the gBRCA mutation in breast cancer. Since the dimensions are too large for slide-based training, we divided WSI into smaller tiles with the original resolution. The tile-based classification was then combined by adding the positive classification result to generate the combined slide-based accuracy. Models were trained based on the annotated tumor location and gBRCA mutation status labeled by a designated breast cancer pathologist. Four models were trained on tiles cropped at 5×, 10×, 20×, and 40× magnification, assuming that low magnification and high magnification may provide different levels of information for classification.

**Results:**

A trained model was validated through an external dataset that contains 17 mutants and 47 wilds. In the external validation dataset, AUCs (95% CI) of DL models that used 40×, 20×, 10×, and 5× magnification tiles among all cases were 0.766 (0.763–0.769), 0.763 (0.758–0.769), 0.750 (0.738–0.761), and 0.551 (0.526–0.575), respectively, while the corresponding magnification slides among all cases were 0.774 (0.642–0.905), 0.804 (0.676–0.931), 0.828 (0.691–0.966), and 0.635 (0.471–0.798), respectively. The study also identified the influence of histological grade to the accuracy of the prediction.

**Conclusion:**

In this paper, the combination of pathology and molecular omics was used to establish the gBRCA mutation risk prediction model, revealing the correlation between the whole-slide histopathological images and gRCA mutation risk. The results indicated that the prediction accuracy is likely to improve as the training data expand. The findings demonstrated that deep CNNs could be used to assist pathologists in the detection of gene mutation in breast cancer.

## Introduction

Female breast cancer (BC) made up 11.7% of 19.3 million new cancer cases in 2020 and has overtaken lung cancer as the most diagnosed cancer globally, and ranks as the fourth leading cause of cancer-related mortality, according to a report from the International Agency for Research on Cancer (Sung et al., 2021). BC is a heterogeneous collection of diseases with various incidences, risk factors, genetic, prognosis, and treatment responses. Genetic susceptibilities to BC may be associated with mutations in a specific gene or a series of genes, including the key tumor suppressor gene BRCA (BRCA1 or BRCA2). BRCA1/2 mutation may be inherited (germline, gBRCA) or may arise *de novo* because of a combination of genetic and environmental factors (somatic) (Engel and Fischer, 2015). The frequency of these genetic mutations varies among different countries and ethnic groups. A study of a large cohort of a Chinese population shows that the BRCA mutation rate was 9.1% in BC patients with at least one risk factor, 3.5% in sporadic patients, and 0.38% in healthy controls (Lang et al., 2017). BRCA1/2 plays an essential role in DNA damage response, DNA double-strand break, repair, transcriptional regulation, etc. Loss of BRCA1/2 educes impairment of the homologous recombination DNA repair pathway, thereby leading to genomic instability which may ultimately contribute to cancer development. Patients who carry a germline pathogenic mutation in the BRCA1/2 gene have a significantly increased risk of developing BC and other cancers (e.g., ovarian, pancreatic, and prostate cancer) (Paul and Paul, 2014). Previous meta-analyses of published trials show that BRCA1 and BRCA2 carriers have a 57–65% and 45–49% probability of developing BC over lifetime, respectively. Furthermore, if there is a positive family history of BC, this risk increases to 85 and 84%, respectively (Antoniou et al., 2003; Chen and Parmigiani, 2007).

BCs with BRCA1/2 mutations are different from sporadic BC in clinical and pathological features. Patients with gBRCA1 mutations have a higher prevalence of triple-negative (absence of estrogen receptor, progesterone receptor, and HER-2 expression), invasive ductal carcinoma with medullary features (Sønderstrup et al., 2018). The multivariate analysis revealed that morphological features predictive of the BRCA1 phenotype include the presence of lymphocytic infiltrate, higher mitotic figures, and pushing margins compared with sporadic BC (Atchley et al., 2008). BRCA2 tumors are also more frequently higher histological grade compared with sporadic tumors. However, the unique characteristic that is significant for BRCA2-associated BC is lack of tubule formation and pushing margins (Atchley et al., 2008). The detection of a pathogenic gBRCA mutation in a woman diagnosed with BC may affect her current cancer treatment and prognosis, but it can also prevent future cancers and identify healthy mutation carriers in their family members (Metcalfe et al., 2014; Faraoni and Graziani, 2018; Torrisia et al., 2019). Knowing one’s gBRCA status plays an important role for healthy women, because cancer can be prevented by risk-reducing mastectomy and salpingo-oophorectomy (Domchek et al., 2010). The latest recommendations in the guidelines for the treatment of gBRCA-mutated advanced BC highlight the promise of platinum-based chemotherapies and poly adenosine diphosphate–ribose polymerase inhibitors (PARPi) (National Comprehensive Cancer Network, 2020). Consequently, genetic testing becomes more and more important to identify patients with gBRCA-mutant tumors.

Although the methodology of detecting genetic variants has greatly improved, molecular testing is usually time-consuming and could be limited by availability of adequate samples. Moreover, the cost of genetic testing is still too high for most families. Therefore, BRCA detection has traditionally been limited to BC patients who have an *a priori* high risk of being a mutation carrier. These risk factors include triple-negative BC, young age at diagnosis (below 45 years), or a family history of breast and/or ovarian cancer (Wong-Brown et al., 2015; Grindedal et al., 2017). Although many guidelines in various countries focus on identifying such high-risk groups, the latest guidelines adopt broader criteria regardless of family history. This supports the increasing evidence in the literature that clinical criteria (e.g., family history) may omit individuals with BRCA1/2 mutations, some of which suggest that BRCA testing should be expanded to a wider population. Thus, the method to predict gene mutations quickly and inexpensively from histopathology images could be beneficial to the treatment of patients with BC given the importance and impact of these mutations.

The latest development in artificial intelligence (AI) provided a novel method to assist clinicians to classify medical information and images (Bera et al., 2019; Bi et al., 2019). The possibility of digitizing whole-slide images (WSIs) of pathology tissue has led to the emergence of AI and machine learning (ML) tools in digital pathology, which can mine the subvisual morphometric phenotypes and ultimately enhance patient management. Recently, pathologists and computer scientists have come together to apply the latest AI technology (e.g., deep learning) to the problem of analyzing pathology slides for assisting diagnosis, prediction, prognosis, and other clinically related purposes, as well as other applications such as improving the efficiency of the diagnostic workflow. In breast pathology, deep learning (DL) has already been applied in classifying the type and subtype of breast tumors, identifying metastasis in lymph nodes, detecting tubular formation and nuclear pleomorphism, tumor grading, counting mitotic figures, etc. (Bejnordi et al., 2017; Sudharshan et al., 2019; Mahmood et al., 2020; Xu et al., 2020). Furthermore, researchers investigated whether the molecular characteristics of cancer are encoded in histomorphological structures that are beyond human apprehension (Xu et al., 2019; Schmauch et al., 2020; Bilal et al., 2021). As such, Shamai et al. (2019) applied an ML method, termed morphological-based molecular profiling (MBMP), on BC specimens to explore the associations between histomorphological characteristics and expression of multiple molecular biomarkers. For at least half of the patients in this study, MBMP seemed to predict the expression of biomarkers and is not inferior to immunohistochemistry (Shamai et al., 2019). Similarly, Narula et al. (2018) trained a deep convolutional neural image processing network to automatically classify histopathological subtypes from digital pathology slides of lung specimens and predict common mutant genes in lung adenocarcinoma.

These results suggest that DL models can be used to effectively assist pathologists in detecting gene mutations and tumor histological subtypes. However, it remains unclear whether DL can be applied to predict BRCA gene mutation status using BC digital pathology slides. Therefore, we focused on the BC specimens and tested whether DL can be trained to predict gBRCA1/2 mutations using images as the only input. In this study, we constructed DL models based on convolutional neural networks (CNN) using WSIs of hematoxylin and eosin (H&E)-stained digital pathology slides obtained from the Jiangsu Province Hospital of Chinese Medicine (JSPHCM) and Jiangsu Cancer Hospital (JSCH) to predict the gBRCA1/2 mutation status in BC.

## Materials and Methods

### Study Cohort

All the cases were collected from two medical centers in China, which were Jiangsu Province Hospital of Chinese Medicine (JSPHCM) and Jiangsu Cancer Hospital (JSCH), Nanjing. A total of 22 BC patients were eventually enrolled in the BRCA-mutation group, and 40 patients were enrolled in the BRCA-wild group. We combined H&E-stained WSIs from two datasets: the JSPHCM dataset, which contains 60 H&E images from 12 BRCA-mutation patients and 50 H&E images from 10 BRCA-wild patients, and the JSCH dataset, which contains 25 H&E images from 10 BRCA-mutation patients and 87 H&E images from 30 BRCA-wild patients. Slides were digitized with a NanoZoomer Digital slide scanner (Hamamatsu Photonics Scientific Instrument Co., Ltd., Beijing, China) at a resolution of ×40. This study has been approved by the Institutional Ethical Review Boards of JSPHCM with patient consent.

The tumor pathology for all patients with BC was reviewed under the criteria of the World Health Organization Classification of Tumors: Breast Tumors (5th edition) (WHO, 2019) by one of our designated breast pathologists. All 22 patients with BRCA mutation have invasive breast carcinoma, not otherwise specified (invasive ductal carcinoma). Among the 40 patients with BRCA wild type, 36 cases were invasive ductal carcinoma, two cases were mucinous carcinoma, one case was invasive lobular carcinoma, and one case was a metaplastic carcinoma. Using Automated Slide Analysis Platform (ASAP 1.9), pathologists can navigate WSI images at a very high resolution and annotate the whole-tumor regions within slides for ease of adjudication. The DL model was trained based on the annotated tumor location.

Pathologists classified all the invasive BCs according to the Nottingham histological grading system (NGS). The NGS has three parameters, which are tubule formation, nuclear pleomorphism, and mitotic count. Each parameter has been divided into three categories, with the score from 1 to 3, assigned as follows: tubule formation (1: 75%, 2: 10–75%, 3: 10%); nuclear pleomorphism (1: none, 2: moderate, 3: pronounced); and the number of mitoses/10 high-power fields (HPF) (40 objective lens) (1: 0–9 mitoses; 2: 10–19 mitoses; and 3: > 19 mitoses). The final histological grade is based on a sum of the scores of the three parameters: 3, 4, or 5 = grade 1; 6 or 7 = grade 2; and 8 or 9 = grade 3 (WHO, 2019). In the cohort of 62 patients, grade 1 tumors have been observed in 1 patient with BRCA-wild (1/40, 2.5%), and none has been observed in patients with BRCA-mutation (0/22, 0%); grade 2 tumors have been observed in 14 patients with BRCA-wild (14/40, 35%) and 3 patients with BRCA-mutation (3/22, 13.6%); and grade 3 tumors have been observed in 25 patients with BRCA-wild (25/40, 62.5%) and 19 patients with BRCA-mutation (19/22, 86.4%).

Data on BRCA1/2 mutations were routinely collected and extracted in clinic from electronic medical records. BRCA testing has been done in a centralized clinical testing center (Nanjing Geneseeq Technology Inc., Nanjing, China), using germline DNA (from blood), according to protocols reviewed and approved by the ethical committee of each participating hospital, and the test results were categorized as either positive or negative of a deleterious mutation.

### Method of DL With Convolution Neural Networks

**FIGURE 1:**
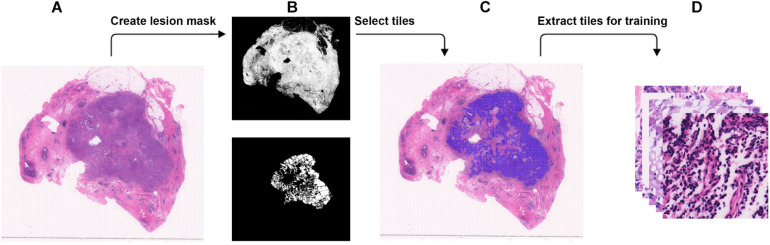
Steps to extract small tiles from whole-slide images at ×40 magnification for training. **(A)** An example WSI with BRCA-mutation or BRCA-wild. **(B)** The binary mask (top) was created using color differences in RGB color space, and then element-wise matrix multiplication was done with the manually labeled lesion (bottom) to create a lesion mask without void spaces. **(C)** Tiles with resolution of 384 × 384 were extracted from the lesion mask. **(D)** The tiles that were extracted will be used in the training.

The DL model we used in this study is a residual neural network (ResNet), which is a type of artificial neural network that builds based on pyramid cells in the cerebral cortex. The typical ResNet is built by having layer-skipping connections to avoid the problem of gradient vanishing. Thus, it allows to train on a deeper neural network (He et al., 2016). In this case, the network is ideal to be used to classify complex histomorphological structures. A ResNet with 18 layers has been used (Figure 1). At the end of the network, a fully connected layer is added for binary classification between BRCA-wild and BRCA-mutation. The model has been trained by using a dual GPU setup with 2 × 1,080 ti graphics card from Nvidia. The stochastic gradient descent method based on adaptive estimation of first-order and second-order moments has been used as the loss function in the training (Kingma and Ba, 2015).

From the JSPHCM dataset reviewed by our designated breast pathologists, 58 H&E images of BRCA-mutation and 44 H&E images of BRCA-wild have been selected for the training, among which 56 H&E images of BRCA-mutation, and 33 H&E images of BRCA-wild are categorized as histological grade 3. From the JSCH dataset, 17 H&E images of BRCA-mutation and 47 H&E images of BRCA-wild have been selected for external validation, among which 17 H&E images of BRCA-mutation and 27 H&E images of BRCA-wild are categorized as histological grade 3, In all the selected images, a brief location of the tumor is annotated and will be used as labels for supervised training. Training and internal testing datasets were created from JSPHCM dataset, and the external testing was created from the JSCH dataset.

### Training Data Preparation

**FIGURE 2:**
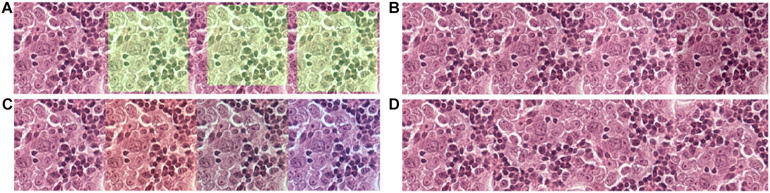
Data augmentation to normalize the training data and simulate and prevent overfitting during training. **(A)** 256 × 256 tiles were randomly extracted from the 320 × 320 tiles. **(B)** The brightness of the tiles was randomly adjusted in a range of –20 to + 20-pixel value in an 8-bit space. **(C)** The red and blue channels of the tiles were randomly adjusted in a range of –20 to + 20-pixel value in an 8-bit space. **(D)** Tiles were randomly flipped up/down and left/right.

WSI has a large resolution which sometimes has a resolution larger than 50,000 × 50,000 pixels. It is not possible to process the entire image for DL due to the memory usage. Therefore, we chose to break down each image into tiles with a smaller resolution (Dimitriou et al., 2019). Using brief annotation of the tumor annotated by designated breast pathologists, tiles with tumor tissue were extracted using the labeled data. To avoid extracting tiles from the void area, a binary mask for cellular tissue was created by using the color spacing in the RGB space. An element-wise matrix multiplication between the binary mask and the labeled tumor area was performed to extract the tumor mask from the binary mask without the void area. The tumor mask was then divided into tiles. For each tile, at least 30% of the area is covered with tissue to make sure no void area is used in the DL computation. A detailed illustration is found in Figure 2. The input size of the DL model is 256 × 256, but during data preparation, we extracted tiles at a resolution of 384 × 384 to create enough resolution space for augmentation during training. To maintain an even number of tiles from each slide, the number of tiles to be extracted from each slide was determined by the minimum number of the tiles that could be extracted among all the slides.

WSI can be inspected at different magnifications. The pixel information varies at different magnifications within a fixed pixel area. Morphological structures of the cellular tissues were preserved at low magnification while better details of cellular structure were preserved at high magnification. Different morphological structures might contain different features that could contribute differently to the DL algorithm. In our study, we used four types of magnifications ranging between ×5, ×10, ×20, and ×40 to find the optimal range of magnification to achieve the best prediction. From 102 slides from the JSPHCM dataset, a total of 18,109 tiles were extracted with 10,140 BRCA-mutation tiles and 7,969 BRCA-wild tiles at ×5 magnification. At ×10 magnification, a total of 58,745 tiles were extracted with 32,344 BRCA-mutation tiles and 26,401 BRCA-wild tiles. At ×20 magnification, a total of 239,108 tiles were extracted with 131,467 BRCA-mutation tiles and 107,641 BRCA-wild tiles. At ×40 magnification, a total of 962,868 tiles were extracted, with 529,242 BRCA-mutation tiles and 433,626 BRCA-wild tiles. All the tiles were randomly and equally divided into 90 and 10% for the training and internal testing datasets, respectively, to be used to train the model. All the tiles with histological grade 3 were labeled.

### Data Augmentation and Training

**FIGURE 3:**
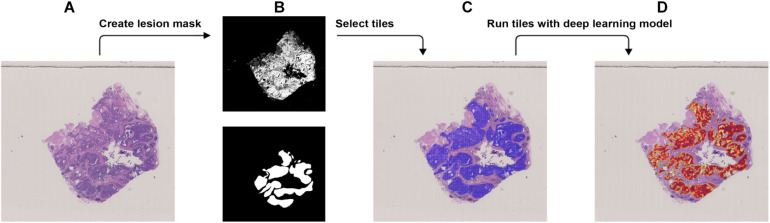
Procedure to validate the trained model using the external dataset with BRCA-mutation. **(A)** The WSI with BRCA-mutation. **(B)** The binary mask (top) was created using color differences in the RGB color space and then element-wise matrix multiplication was done with the manually labeled lesion (bottom) to create a lesion mask without void spaces. **(C)** Tiles with a resolution of 256 × 256 were extracted from the lesion mask. **(D)** Heatmap which illustrates the probability of the region being classified as BRCA-mutation.

Due to limitations among the slides available, to prevent overfitting of the model, each tile in the training dataset undergoes multiple steps of augmentation before feeding into the model (Figure 3). Tiles at a resolution of 256 × 256 were extracted randomly from tiles created during data preparation that has a 384 × 384 resolution. The extracted tiles later went through random flips in left/right and up/down orientation to increase data complexity. Since each slide has variability in artifacts and staining, and H&E staining has high pixel intensity in the red and blue channels with RGB color space, a random intensity adjustment at both red and blue channels was also done in a range of –20 to + 20-pixel value in the 8-bit color channel to synthetically capture the variabilities. In the end, an overall brightness change was added in a range of –20 to + 20 to the image. Through these extensive augmentations, we tried to increase the data complexity to let the model focus on cellular morphology or other “unknown” features to differentiate BRCA-mutation and BRCA-wild, without being influenced by the color saturation or brightness, which is different case by case due to scanning, staining method, etc. Models were trained for extensiveness among iterations until the minimum loss function gradient of the training validation dataset is reached. The same steps were repeated for tiles with histological grade 3.

### External Validation

**FIGURE 4:**
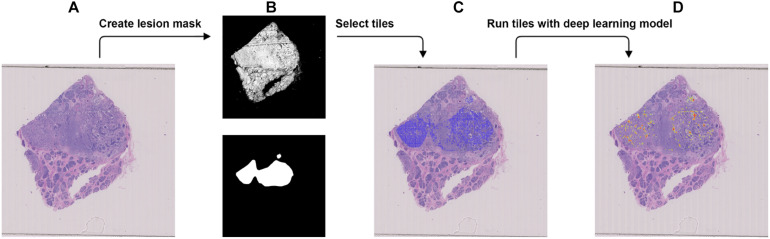
Procedure to validate the trained model using the external dataset with BRCA-wild. **(A)** The WSI with BRCA-wild. **(B)** The binary mask (top) was created using color differences in the RGB color space and then element-wise matrix multiplication was done with the manually labeled lesion (bottom) to create a lesion mask without void spaces. **(C)** Tiles with a resolution of 256 × 256 were extracted from the lesion mask. **(D)** Heatmap which illustrates the probability of the region being classified as BRCA-mutation.

**FIGURE 5:**
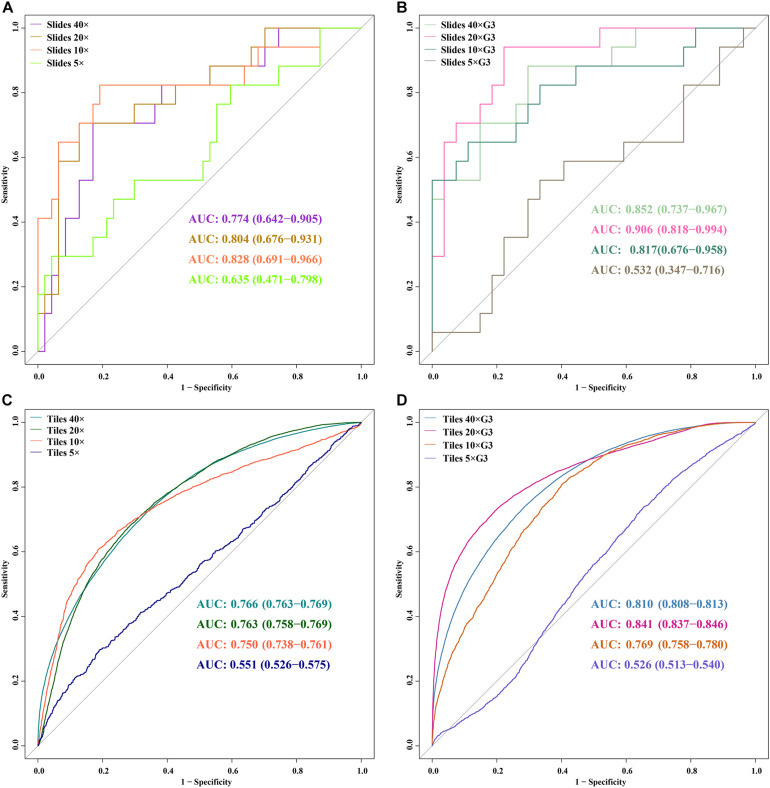
ROC curves of 5 DL models for slides and tiles at ×40, ×20, ×10, and ×5 magnification. **(A)** slides, **(B)** slides G3, **(C)** tiles, **(D)** tiles G3.

**FIGURE 6:**
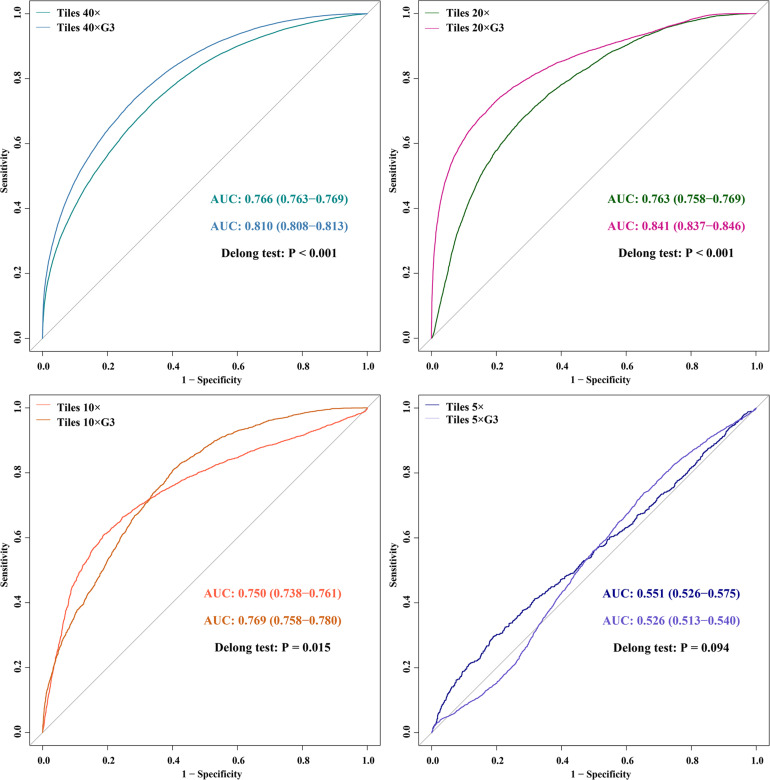
The ROC and comparison of AUCs of DL models using tiles between all cases and G3 cases.

**FIGURE 7:**
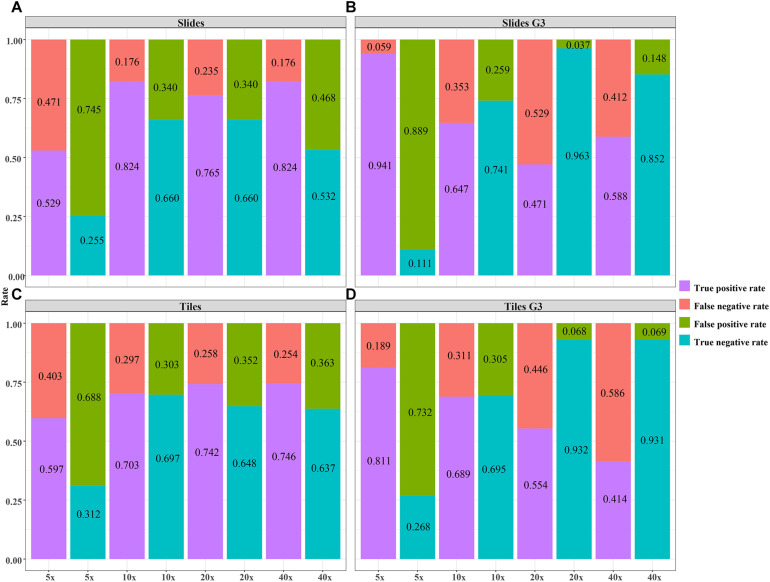
The validity of the DL model using different magnification tiles and slides. **(A)** slides, **(B)** slides G3, **(C)** tiles, **(D)** tiles G3.

We used the JSCH dataset for external validation since it was not involved in any training. It served as a good validation dataset to evaluate the performance and robustness of our trained DL model. During external validation, each image was broken down into 256 × 256 tiles from the binary mask and the labeled tumor mask, which was the same as the data preparation for training using the range of ×5, ×10, ×20, and ×40 magnifications. The extracted tiles were fed into the model as the input and model output classification probability of BRCA-mutation. The outputs were illustrated as heatmap images; the higher the probability of BRCA-mutation, the higher the heatmap intensity (Figures 4, 5). The average probability across all the slides was calculated from the probability of the tiles. Anything higher than the 0.5 probability was considered as BRCA-mutation. The receiver operating characteristic curve (ROC), area under the ROC (AUC), and confusion matrix were created for the testing results (Figures 6, 7 and Table 1). The same steps were repeated for WSI with histological grade 3.

**TABLE 1:** The likelihood ratio (+LR and −LR) and predictive value (PPV and NPV) of DL models.

**Classification**	**+LR**	**−LR**	**PPV**	**NPV**
Slides × 40	1.761	0.331	0.389	0.893
Slides × 20	2.250	0.356	0.448	0.886
Slides × 10	2.424	0.267	0.467	0.912
Slides × 5	0.710	1.847	0.205	0.6
Slides × 40 G3	3.973	0.484	0.714	0.767
Slides × 20 G3	12.730	0.549	0.889	0.742
Slides × 10 G3	2.498	0.476	0.611	0.769
Slides × 5 G3	1.058	0.532	0.400	0.750
Tiles × 40	2.055	0.399	0.567	0.797
Tiles × 20	2.108	0.398	0.571	0.799
Tiles × 10	2.320	0.426	0.585	0.794
Tiles × 5	0.868	1.291	0.339	0.567
Tiles × 40 G3	6.000	0.629	0.817	0.681
Tiles × 20 G3	8.147	0.478	0.859	0.736
Tiles × 10 G3	2.259	0.447	0.624	0.752
Tiles × 5 G3	1.108	0.705	0.449	0.658

*+LR, positive likelihood ratio; −LR, negative likelihood ratio; PPV, positive predictive value; NPV, negative predictive value; G3, grade 3.*

### Statistical Analysis

We performed the ROC and calculated the validity (true positive rate, false negative rate, false positive rate, true negative rate, likelihood ratio) and predictive value to demonstrate the classification ability of the DL model. Delong tests were then applied to compare the AUC of slides and tiles with different magnifications from all cases and grade 3 cases. A percentage bar plot was plotted to visualize the validity (true positive rate, false negative rate, true negative rate, and false positive rate) of the DL model. Box plot and Student’s *t*-test were used to compare the predictive BRCA mutation probability of mutation and wild group by the DL model. A Bland–Altman plot was plotted to evaluate the agreement of predicted mutation probability for per-slide at different magnifications. All statistical analyses and figures were performed by using R version 4.0.3 (The R Foundation for Statistical Computing; Vienna, Austria) with packages “ggplot2” and “ggthemes.” A *p*-value of less than 0.05 was considered as statistical significance.

## Results

In the external validation dataset, AUCs (95% CI) of DL models using ×40, ×20, ×10, and ×5 magnification tiles among all cases were 0.766 (0.763–0.769), 0.763 (0.758–0.769), 0.750 (0.738–0.761), and 0.551 (0.526–0.575), respectively; those using corresponding magnification tiles among grade 3 cases were 0.810 (0.808–0.813), 0.841 (0.837–0.846), 0.769 (0.758–0.780), and 0.526 (0.513–0.540), respectively, those using corresponding magnification slides among all cases were 0.774 (0.642–0.905), 0.804 (0.676–0.931), 0.828 (0.691–0.966), and 0.635 (0.471–0.798), respectively, and those using corresponding magnification slides among grade 3 cases were 0.852 (0.737–0.967), 0.906 (0.818–0.994), 0.817 (0.676–0.958), and 0.532 (0.347–0.716), respectively (Figure 5). Delong test demonstrated that AUCs (95% CI) of DL models using ×40 (*P* < 0.001), ×20 (*P* < 0.001), and ×10 (*P* < 0.001) magnification tiles among all cases were less than those among grade 3 cases, and that using ×5 magnification tiles among all cases and grade 3 cases was marginally significant (Figure 6). Additionally, the ROC and the comparison of AUCs among another magnification slides and tiles are listed in Supplementary Figure 1.

**FIGURE 8:**
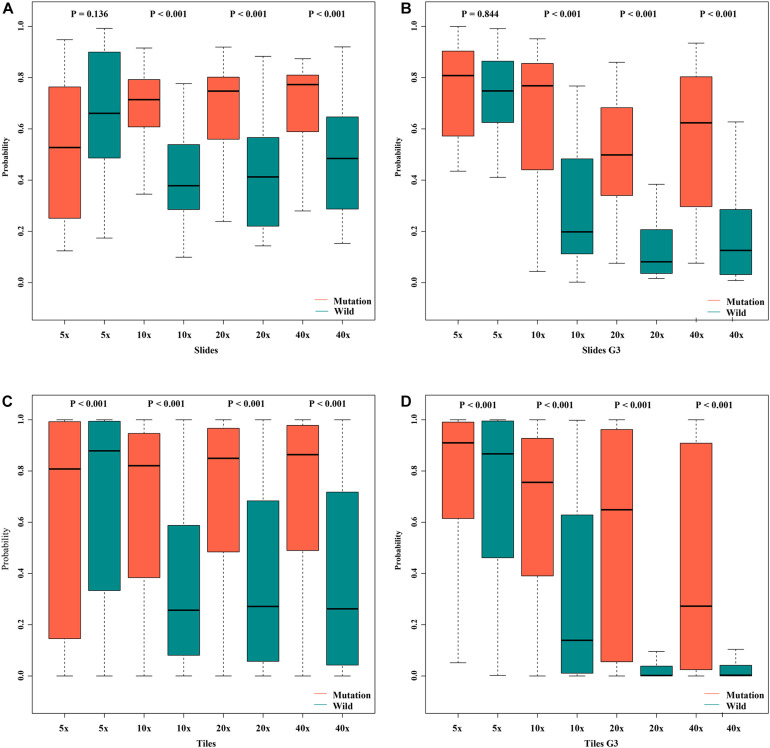
Box plot of probabilities on slide and tile level with different magnifications. **(A)** slides, **(B)** slides G3, **(C)** tiles, **(D)** tiles G3.

The validity and predictive value of DL models using different magnification tiles or slides are listed in Figure 8 and Table 1. The positive likelihood ratios (+ LR) of DL models using ×10, ×20, and ×40 magnification slides and tiles among all cases and grade 3 cases were high, and the negative likelihood ratios (-LR) were low. Meanwhile, the almost negative predictive values of those were more than 0.700, except ×40 magnification tiles among grade 3 cases (0.681). The above results suggest that the validity of these models was high, and a higher proportion of patients with negative diagnoses (wild rather than mutation) were actually negative. Slides at ×10 magnification that had the best performance suggest that a bigger field of view contributes positively to the classification between BRCA-mutation and BRCA-wild. It corresponds to the features for the prediction of BRCA1 and BRCA2 mutation, such as the presence of lymphocytic infiltrate, pushing margin, and lack of tubule formation, which are mostly shown in ×10 slides rather than ×20 and ×40 slides. Moreover, slides at ×20 magnification had the best performance among grade 3 cases, which suggests that a higher histological grade of BC has more complex and tiny features. Therefore, an appropriate higher magnification enables the DL model to have better ability of gBRCA-mutation classification.

**FIGURE 9:**
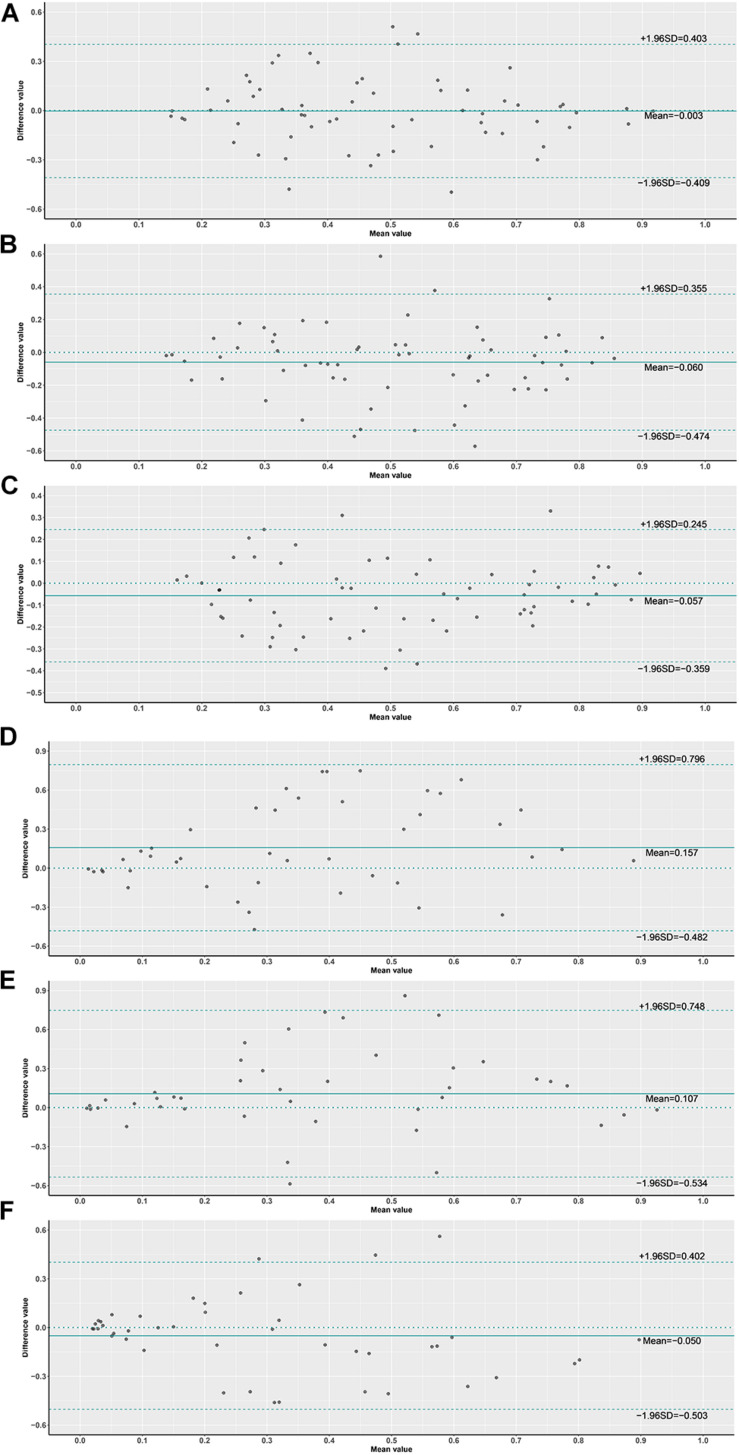
Bland–Altman plot of probabilities on slide level. **(1) (A)** ×10 vs. ×20 slides, **(B)** ×10 vs. ×40 slides, **(C)** ×20 vs. ×40 slides, **(2) (D)** ×10 vs. ×20 slides G3, **(E)** ×10 vs. ×40 slides G3, **(F)** ×20 vs. ×40 slides G3.

In order to assess the classification accuracy on per-slide level and per-tile level, the results were aggregated using ×40, ×20, ×10, and ×5 magnifications. The ×10, ×20, and ×40 magnifications that were applied to the mutation groups show significantly higher probabilities than the wild group in both slide level and tile level (Figure 8). Next, according to the results of the box plot and the comparison, we plotted the Bland–Altman plot using the predictive probability obtained from the three magnifications (×10, ×20, and ×40) to investigate the agreement of per-slide on classification among all cases and grade 3 cases. Figures 9-1, 9-2 show that most of the points were distributed within the range of 95% limits of agreement (LoA). In other words, all results were in a good agreement.

## Discussion

We have developed a computerized system (CNN-based DL) to predict molecular markers (gBRCA mutation) of BC by analysis of tumor histomorphology. Since BRCA1/2 gene mutations occur at a relatively high frequency in BC, to predispose an individual with developing BC and other cancers, PARP inhibitors are regarded as one of the potential targeted drugs for gBRCA mutant BC. Although it has been suggested that gene mutations could be predicted from H&E-stained WSIs [AUC of ∼0.85 for the prediction of STK11 mutations from lung cancer H&E images (Narula et al., 2018); AUC of ∼0.71 for the prediction of SPOP mutations from prostate cancer H&E images (Schaumberg et al., 2018); AUC of 0.71∼0.89 for the prediction of CTNNB1, FMN2, TP53, and ZFX4 mutations from hepatocellular carcinoma H&E images (Chen et al., 2020)], prior to this study, it was unclear whether gBRCA mutations would affect the pattern of tumor cells on BC H&E-stained WSIs.

We have trained the DL model (ResNet) using the presence or absence of BRCA mutation as a label revealed that gBRCA mutational status can be predicted from image data alone (AUCs 0.55–0.91 in different DL models). Interestingly, BC cases with high-grade histology (Grade 3) achieved higher AUC (95% CI) using the DL model at ×40, ×20, and ×10 magnifications. It corresponds to that gBRCA-mutated BCs are more frequently of higher histological grade. Moreover, slides at ×20 magnification had the best performance among grade 3 cases (AUC up to 0.906), and slides at ×10 magnification had the highest negative predictive value (0.91), which suggest that histopathological images with different magnifications can represent different information. The images with low magnification (×5–×10) cover a larger field of view, while the images with high magnification (×20–×40) correspond to a relatively small area with more details. In the analysis of histopathological images, it is necessary to recognize complex morphological patterns of various sizes. AI can capture cellular level information by high-magnification images and tissue spatial structures by low-magnification images at the same time. We found that the tumor morphology captured in H&E-stained images contains signals that predict the status of tumor molecular markers. DL approaches can extract sub-visual morphological phenotypes from WSIs beyond that which a human is capable. We show that DL can recognize a group with morphological features within the tissue structure captured from WSIs and predict the gBRCA1/2 mutation status. The prediction of gBRCA mutation using DL will be of great significance for select patients who are most likely to respond to PARP inhibitor-targeted therapy and identification of healthy mutation carriers within their families.

The model used in this study is ResNet with 18 layers. It is a simplified ResNet with a smaller number of layers, compared with the original ResNet (He et al., 2016) proposed by Kaiming He. The advantage of ResNet is that it is possible to go deeper without losing generalization capability. It is making sense that the deeper the network, the better the result for the convolutional network. However, we need to simplify the network to avoid overfitting due to the size of the dataset. The depth of the model can be adjusted as the size of datasets grows. Both models trained at ×10 and ×40 magnification show effective results. Each model operates at a different level of magnification, which the user could choose to use a single model for efficiency or a combined model for higher accuracy. With the updates in the computer systems and hardware, the resolution of the tiles and the number of models at multiple magnifications could also be improved to exploit for better accuracy.

This is the first study to predict the BRCA gene mutation in BC, while using an independent database from JSCH to externally validate the performance of the model. It has been proved that CNN-based DL can be used to assist gene mutation prediction based on histopathological slides in BC. However, the present study has several limitations. One limiting factor in achieving higher accuracy lies in the small number of slides containing BRCA mutation instances that can be used for training and validation. Furthermore, the ability of any such AI approaches to predict all targetable mutations is critical, as more and more molecular markers are expected to be quantified in each sample, and treatment decisions are usually delayed until information about all such driver mutations is obtained. Subsequently, further validation of our model is necessary in a larger dataset with multiple centers and other BC-related genes should be considered in further study.

In conclusion, the study demonstrates that CNN-based DL can predict the gBRCA mutation status from H&E-stained WSIs in BC, and DL potential to improve cancer prognosis and therapeutics by utilizing biological markers currently imperceptible to clinicians. Although AI cannot completely replace humans in practice nowadays, gene mutation prediction can be used as a prescreening to improve the cost efficiency before next-generation sequencing, thereby improving the performance of precision medicine.

## Data Availability Statement

The original contributions presented in the study are included in the article/Supplementary Material, further inquiries can be directed to the corresponding author/s.

## Ethics Statement

The studies involving human participants were reviewed and approved by the Ethics Committee in Affiliated Hospital of Nanjing University of Chinese Medicine (Jiangsu Provincial Hospital of Chinese Medicine). The ethics committee waived the requirement of written informed consent for participation.

## Author Contributions

LX and YFZ: conception and design. XW, YZ, FK, JC, XX, and YFZ: provision of study materials or patients. XW, CZ, XL, CW, and LX: collection and assembly of data. XW, WW, and DW: data analysis and interpretation. All authors: writing and final approval of the manuscript.

## Conflict of Interest

The authors declare that the research was conducted in the absence of any commercial or financial relationships that could be construed as a potential conflict of interest. The reviewer RY declared a shared affiliation with the authors YFZ and XW to the handling editor at the time of review.

## Supplementary Material

Supplementary Figure 1 The ROC and the comparison of AUCs among another magnification at tiles.
