# Supplementary figures and images for: Prediction of BRCA Gene Mutation in Breast Cancer Based on Deep Learning and Histopathology Images
Source: Front Genet. 2021 Jul 20;12:661109. doi: 10.3389/fgene.2021.661109 (PMC8329536; doi:10.3389/fgene.2021.661109)

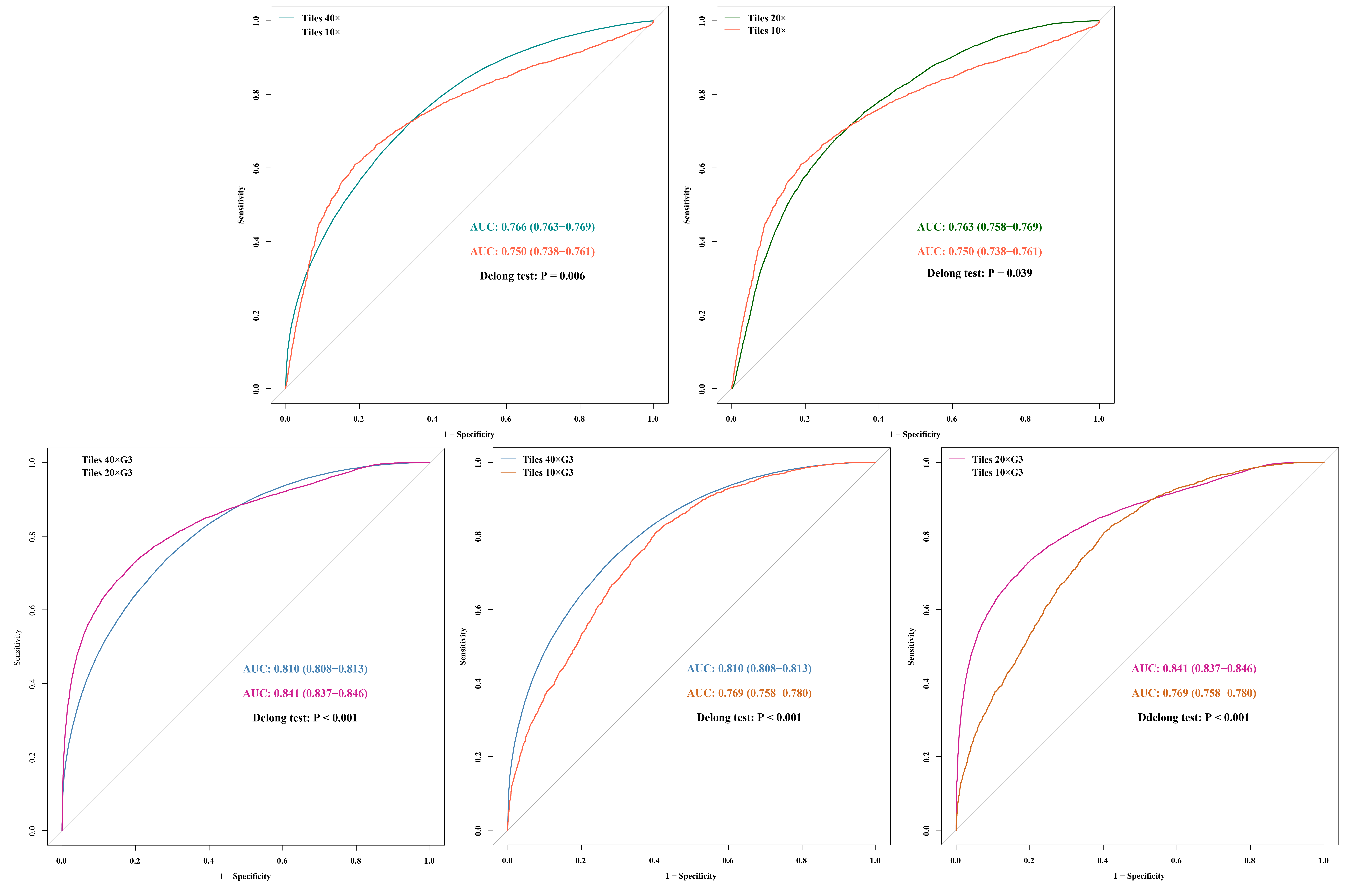

Supplement: Supplementary Figure 1 — The ROC and the comparison of AUCs among another magnification at tiles. [file Image_1.TIF]
